# 12-(4-Chloro­phen­yl)-9,9-dimethyl-9,10-dihydro-8*H*-benzo[*a*]xanthen-11(12*H*)-one

**DOI:** 10.1107/S1600536810004125

**Published:** 2010-02-06

**Authors:** De-Ling Li, Li-Hong Wang

**Affiliations:** a Department of Chemistry, Tangshan Normal College, Tangshan 063000, People’s Republic of China

## Abstract

The title compound, C_25_H_21_ClO_2_, was synthesized *via* the three-component coupling of 4-chloro­benzaldehyde, 2-naphthol and 5,5-dimethyl­cyclo­hexane-1,3-dione. The pyran ring adopts a boat conformation, while the cyclo­hexenone ring is in an envelope conformation. The 4-chloro­phenyl ring is almost perpendicular to the pyran ring [dihedral angle = 87.39 (1)°]. In the crystal, mol­ecules are connected by inter­molecular C—H⋯O hydrogen bonds.

## Related literature

For the biological activity of xanthenes and benzoxanthenes, see: Poupelin *et al.* (1978 ▶); Lambert *et al.* (1997 ▶) and for their applications see: Ion *et al.* (1998 ▶); Saint-Ruf *et al.* (1975 ▶).
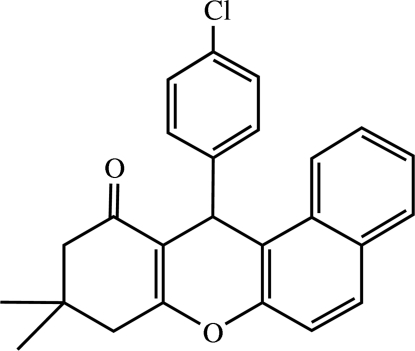


## Experimental

### 

#### Crystal data

C_25_H_21_ClO_2_
*M* *_r_* = 388.87
Monoclinic, 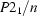

*a* = 10.293 (2) Å
*b* = 11.621 (2) Å
*c* = 16.447 (3) Å
β = 90.04 (3)°
*V* = 1967.3 (7) Å^3^
*Z* = 4
Mo *K*α radiation
μ = 0.21 mm^−1^
*T* = 113 K
0.16 × 0.14 × 0.08 mm

#### Data collection

Rigaku Saturn CCD area-detector diffractometer
Absorption correction: multi-scan (*CrystalClear*; Rigaku/MSC, 2005 ▶) *T*_min_ = 0.967, *T*_max_ = 0.983
15871 measured reflections
4667 independent reflections
2863 reflections with *I* > 2σ(*I*)
*R*_int_ = 0.079

#### Refinement

*R*[*F* ^2^ > 2σ(*F* ^2^)] = 0.053
*wR*(*F* ^2^) = 0.136
*S* = 0.91
4667 reflections
255 parameters
H-atom parameters constrained
Δρ_max_ = 0.43 e Å^−3^
Δρ_min_ = −0.50 e Å^−3^

### 

Data collection: *CrystalClear* (Rigaku/MSC, 2005 ▶); cell refinement: *CrystalClear*; data reduction: *CrystalClear*; program(s) used to solve structure: *SHELXS97* (Sheldrick, 2008 ▶); program(s) used to refine structure: *SHELXL97* (Sheldrick, 2008 ▶); molecular graphics: *SHELXTL* (Sheldrick, 2008 ▶); software used to prepare material for publication: *SHELXTL*.

## Supplementary Material

Crystal structure: contains datablocks I, global. DOI: 10.1107/S1600536810004125/bv2134sup1.cif

Structure factors: contains datablocks I. DOI: 10.1107/S1600536810004125/bv2134Isup2.hkl

Additional supplementary materials:  crystallographic information; 3D view; checkCIF report

## Figures and Tables

**Table 1:** Hydrogen-bond geometry (Å, °)

*D*—H⋯*A*	*D*—H	H⋯*A*	*D*⋯*A*	*D*—H⋯*A*
C24—H24⋯O2^i^	0.95	2.56	3.376 (2)	144

Symmetry code: (i) 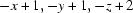.

**Fig. 1.**
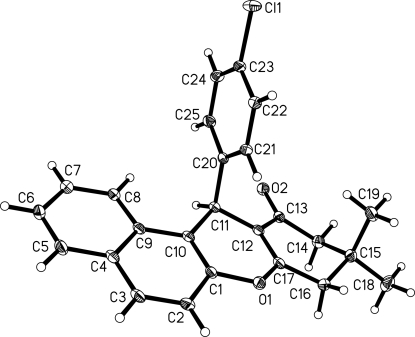
A view of the molecular structure of (I), showing the atom-numbering scheme. Dispacement ellipsoids are drawn at the 30% probability level.

**Fig. 2.**
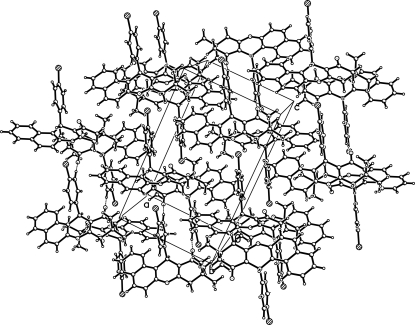
The packing, showing hydrogen-bond interactions as dashed lines, H atoms are shown as small spheres of arbitary radii.

## supplementary crystallographic information

### Comment

Xanthenes and benzoxanthenes are important biologically active heterocycles. They possess anti-inflammatory (Poupelin *et al.*, 1978) and antiviral (Lambert *et al.*, 1997) activities. These compounds are utilized as antagonists for paralyzing action of zoxazolamine (Saint-Ruf *et al.*,1975) and in photodynamic therapy (Ion *et al.*,1998). We report herein the crystal structure of the title compound.

The pyran ring of the title molecule (Fig.1) adopts a boat conformation. The cyclohexenone ring is in an envelope conformation with atom C15 at the flap. The 4-chloropheny ring and the planar part of the pyran ring (C1/C10/C12/C17) are nearly perpendicular to each other, with a dihedral angle of 87.39 (1)°. In the crystal, the molecules are connected by C—H···O hydrogen bonds.

### Experimental

To a mixture of 2-naphthol (1.0 mmol), 4-chlorobenzaldehyde (1.0 mmol), and 5,5-dimethylcyclohexane-1,3-dione (1.1 mmol) was added strontium trifluoromethanesulfonate (0.1 mmol) in 1,2-dichloroethane (2 ml). The mixture was stirred at 80 °C for 5 h. The progress of the reaction was monitored by TLC. After completion of the reaction, water was added and the product was extracted with ethyl acetate (3 x 10 ml). The organic layer was dried (MgSO_4_)and evaporated, and the crude product was purified by flash chromatography on silica gel. Pure product crystallized slowly at room temperature in ethanol. A single-crystal was obtained by slow evaporation of a solution in ethanol.

### Refinement

All H atoms were included in the refinement in the riding model approximation, with C–H = 0.95–1.00 Å, and with *U*_iso_(H) = 1.2 *U*_eq_(C) or 1.5 *U*_eq_(C) for methyl H atoms.

### Crystal data

**Table:**

C_25_H_21_ClO_2_	*F*(000) = 816
*M**_r_* = 388.87	*D*_x_ = 1.313 Mg m^−^^3^
Monoclinic, *P*2_1_/*n*	Mo *K*α radiation, λ = 0.71073 Å
*a* = 10.293 (2) Å	Cell parameters from 5755 reflections
*b* = 11.621 (2) Å	θ = 2.2–27.8°
*c* = 16.447 (3) Å	µ = 0.21 mm^−^^1^
β = 90.04 (3)°	*T* = 113 K
*V* = 1967.3 (7) Å^3^	Prism, colourless
*Z* = 4	0.16 × 0.14 × 0.08 mm

### Data collection

**Table:**

Rigaku Saturn CCD area-detector diffractometer	4667 independent reflections
Radiation source: rotating anode	2863 reflections with *I* > 2σ(*I*)
confocal	*R*_int_ = 0.079
Detector resolution: 7.31 pixels mm^-1^	θ_max_ = 27.9°, θ_min_ = 2.2°
ω and φ scans	*h* = −12→13
Absorption correction: multi-scan (*CrystalClear*; Rigaku/MSC, 2005)	*k* = −15→15
*T*_min_ = 0.967, *T*_max_ = 0.983	*l* = −16→21
15871 measured reflections	

### Refinement

**Table:**

Refinement on *F*^2^	Primary atom site location: structure-invariant direct methods
Least-squares matrix: full	Secondary atom site location: difference Fourier map
*R*[*F*^2^ > 2σ(*F*^2^)] = 0.053	Hydrogen site location: inferred from neighbouring sites
*wR*(*F*^2^) = 0.136	H-atom parameters constrained
*S* = 0.91	*w* = 1/[σ^2^(*F*_o_^2^) + (0.0704*P*)^2^] where *P* = (*F*_o_^2^ + 2*F*_c_^2^)/3
4667 reflections	(Δ/σ)_max_ = 0.001
255 parameters	Δρ_max_ = 0.43 e Å^−^^3^
0 restraints	Δρ_min_ = −0.50 e Å^−^^3^

### Special details

**Table:**

Geometry. All e.s.d.'s (except the e.s.d. in the dihedral angle between two l.s. planes) are estimated using the full covariance matrix. The cell e.s.d.'s are taken into account individually in the estimation of e.s.d.'s in distances, angles and torsion angles; correlations between e.s.d.'s in cell parameters are only used when they are defined by crystal symmetry. An approximate (isotropic) treatment of cell e.s.d.'s is used for estimating e.s.d.'s involving l.s. planes.
Refinement. Refinement of *F*^2^ against ALL reflections. The weighted *R*-factor *wR* and goodness of fit *S* are based on *F*^2^, conventional *R*-factors *R* are based on *F*, with *F* set to zero for negative *F*^2^. The threshold expression of *F*^2^ > σ(*F*^2^) is used only for calculating *R*-factors(gt) *etc*. and is not relevant to the choice of reflections for refinement. *R*-factors based on *F*^2^ are statistically about twice as large as those based on *F*, and *R*- factors based on ALL data will be even larger.

### Fractional atomic coordinates and isotropic or equivalent isotropic displacement parameters (Å^2^)

**Table:**

	*x*	*y*	*z*	*U*_iso_*/*U*_eq_	
Cl1	0.73445 (5)	0.84810 (5)	1.12407 (3)	0.03409 (17)	
O1	0.32484 (13)	0.87906 (11)	0.73299 (7)	0.0238 (3)	
O2	0.25555 (13)	0.57878 (11)	0.92168 (7)	0.0263 (3)	
C1	0.44337 (19)	0.83375 (16)	0.70869 (10)	0.0209 (4)	
C2	0.4964 (2)	0.88806 (17)	0.63881 (10)	0.0245 (4)	
H2	0.4497	0.9473	0.6117	0.029*	
C3	0.6149 (2)	0.85411 (17)	0.61122 (11)	0.0257 (5)	
H3	0.6517	0.8917	0.5654	0.031*	
C4	0.68478 (19)	0.76365 (17)	0.64948 (10)	0.0241 (4)	
C5	0.8075 (2)	0.72738 (18)	0.62115 (11)	0.0290 (5)	
H5	0.8463	0.7659	0.5764	0.035*	
C6	0.8714 (2)	0.63750 (19)	0.65724 (11)	0.0302 (5)	
H6	0.9544	0.6146	0.6378	0.036*	
C7	0.8141 (2)	0.57895 (18)	0.72317 (11)	0.0287 (5)	
H7	0.8577	0.5153	0.7471	0.034*	
C8	0.6959 (2)	0.61332 (17)	0.75303 (11)	0.0244 (4)	
H8	0.6596	0.5739	0.7982	0.029*	
C9	0.62679 (18)	0.70654 (16)	0.71777 (10)	0.0215 (4)	
C10	0.50399 (18)	0.74502 (16)	0.74811 (10)	0.0199 (4)	
C11	0.44318 (18)	0.69161 (16)	0.82395 (10)	0.0202 (4)	
H11	0.4478	0.6059	0.8194	0.024*	
C12	0.30257 (18)	0.72721 (16)	0.82882 (10)	0.0202 (4)	
C13	0.21423 (19)	0.66017 (16)	0.88135 (10)	0.0211 (4)	
C14	0.07188 (19)	0.69208 (17)	0.88032 (11)	0.0261 (4)	
H14A	0.0309	0.6643	0.9311	0.031*	
H14B	0.0292	0.6524	0.8342	0.031*	
C15	0.0482 (2)	0.82193 (17)	0.87251 (11)	0.0260 (5)	
C16	0.11871 (19)	0.86281 (17)	0.79483 (11)	0.0248 (4)	
H16A	0.0684	0.8377	0.7467	0.030*	
H16B	0.1215	0.9480	0.7945	0.030*	
C17	0.25322 (19)	0.81783 (16)	0.78815 (10)	0.0216 (4)	
C18	−0.0967 (2)	0.8461 (2)	0.86518 (14)	0.0378 (6)	
H18A	−0.1110	0.9294	0.8622	0.057*	
H18B	−0.1418	0.8149	0.9128	0.057*	
H18C	−0.1305	0.8095	0.8158	0.057*	
C19	0.1024 (2)	0.88391 (19)	0.94769 (12)	0.0323 (5)	
H19A	0.0891	0.9670	0.9419	0.048*	
H19B	0.1955	0.8677	0.9528	0.048*	
H19C	0.0571	0.8565	0.9964	0.048*	
C20	0.51814 (18)	0.72951 (16)	0.90026 (10)	0.0196 (4)	
C21	0.53737 (19)	0.84537 (16)	0.91539 (10)	0.0217 (4)	
H21	0.5033	0.9007	0.8785	0.026*	
C22	0.60548 (19)	0.88237 (17)	0.98344 (10)	0.0227 (4)	
H22	0.6193	0.9620	0.9930	0.027*	
C23	0.65267 (18)	0.80068 (18)	1.03694 (10)	0.0235 (4)	
C24	0.63531 (19)	0.68441 (18)	1.02455 (11)	0.0261 (4)	
H24	0.6684	0.6298	1.0622	0.031*	
C25	0.5679 (2)	0.64900 (17)	0.95526 (11)	0.0245 (4)	
H25	0.5556	0.5692	0.9454	0.029*	

### Atomic displacement parameters (Å^2^)

**Table:**

	*U*^11^	*U*^22^	*U*^33^	*U*^12^	*U*^13^	*U*^23^
Cl1	0.0361 (4)	0.0441 (4)	0.0221 (3)	−0.0042 (2)	−0.0111 (2)	0.0026 (2)
O1	0.0252 (8)	0.0268 (8)	0.0194 (7)	0.0030 (6)	0.0051 (5)	0.0050 (5)
O2	0.0293 (9)	0.0257 (8)	0.0239 (7)	0.0018 (6)	0.0032 (6)	0.0039 (5)
C1	0.0202 (11)	0.0275 (11)	0.0148 (9)	−0.0006 (8)	0.0025 (7)	−0.0025 (7)
C2	0.0312 (12)	0.0260 (11)	0.0163 (9)	−0.0007 (9)	0.0001 (8)	0.0023 (7)
C3	0.0294 (12)	0.0331 (12)	0.0146 (9)	−0.0039 (9)	0.0043 (8)	0.0002 (7)
C4	0.0267 (11)	0.0302 (11)	0.0154 (9)	−0.0041 (9)	0.0030 (7)	−0.0036 (7)
C5	0.0263 (12)	0.0398 (13)	0.0208 (10)	−0.0025 (9)	0.0071 (8)	−0.0026 (8)
C6	0.0235 (12)	0.0424 (13)	0.0248 (10)	0.0021 (9)	0.0051 (8)	−0.0052 (9)
C7	0.0280 (12)	0.0350 (13)	0.0231 (10)	0.0047 (9)	0.0001 (8)	−0.0011 (8)
C8	0.0241 (11)	0.0320 (12)	0.0172 (9)	0.0012 (9)	0.0014 (7)	−0.0014 (7)
C9	0.0219 (11)	0.0279 (11)	0.0147 (8)	−0.0027 (8)	0.0008 (7)	−0.0039 (7)
C10	0.0224 (11)	0.0234 (11)	0.0138 (8)	−0.0028 (8)	0.0002 (7)	−0.0025 (7)
C11	0.0228 (11)	0.0225 (10)	0.0152 (8)	0.0026 (8)	0.0002 (7)	−0.0007 (7)
C12	0.0222 (11)	0.0242 (10)	0.0143 (8)	−0.0007 (8)	0.0002 (7)	−0.0023 (7)
C13	0.0231 (11)	0.0204 (10)	0.0197 (9)	−0.0007 (8)	−0.0002 (7)	−0.0035 (7)
C14	0.0231 (12)	0.0303 (12)	0.0250 (10)	−0.0031 (9)	0.0033 (8)	0.0015 (8)
C15	0.0243 (12)	0.0297 (12)	0.0240 (10)	0.0035 (9)	0.0039 (8)	0.0020 (8)
C16	0.0235 (12)	0.0300 (11)	0.0210 (9)	0.0035 (9)	−0.0007 (7)	0.0009 (8)
C17	0.0242 (11)	0.0265 (11)	0.0140 (8)	−0.0012 (8)	0.0026 (7)	−0.0023 (7)
C18	0.0275 (13)	0.0428 (15)	0.0432 (13)	0.0074 (10)	0.0068 (10)	0.0099 (10)
C19	0.0425 (15)	0.0304 (12)	0.0240 (10)	0.0051 (10)	0.0072 (9)	−0.0021 (8)
C20	0.0183 (10)	0.0262 (11)	0.0143 (8)	0.0020 (8)	0.0034 (7)	0.0016 (7)
C21	0.0238 (11)	0.0254 (11)	0.0158 (9)	0.0030 (8)	0.0000 (7)	0.0029 (7)
C22	0.0240 (11)	0.0250 (11)	0.0192 (9)	0.0012 (8)	−0.0002 (7)	0.0002 (7)
C23	0.0206 (11)	0.0347 (12)	0.0152 (9)	0.0015 (9)	0.0008 (7)	0.0003 (8)
C24	0.0261 (12)	0.0335 (12)	0.0188 (9)	0.0023 (9)	−0.0012 (8)	0.0070 (8)
C25	0.0293 (12)	0.0242 (11)	0.0199 (9)	0.0015 (9)	0.0019 (8)	0.0026 (7)

### Geometric parameters (Å, °)

**Table:**

Cl1—C23	1.7504 (19)	C13—C14	1.511 (3)
O1—C17	1.369 (2)	C14—C15	1.534 (3)
O1—C1	1.388 (2)	C14—H14A	0.9900
O2—C13	1.231 (2)	C14—H14B	0.9900
C1—C10	1.368 (3)	C15—C18	1.523 (3)
C1—C2	1.420 (3)	C15—C19	1.536 (3)
C2—C3	1.361 (3)	C15—C16	1.545 (3)
C2—H2	0.9500	C16—C17	1.484 (3)
C3—C4	1.420 (3)	C16—H16A	0.9900
C3—H3	0.9500	C16—H16B	0.9900
C4—C5	1.411 (3)	C18—H18A	0.9800
C4—C9	1.435 (2)	C18—H18B	0.9800
C5—C6	1.370 (3)	C18—H18C	0.9800
C5—H5	0.9500	C19—H19A	0.9800
C6—C7	1.410 (3)	C19—H19B	0.9800
C6—H6	0.9500	C19—H19C	0.9800
C7—C8	1.372 (3)	C20—C21	1.383 (3)
C7—H7	0.9500	C20—C25	1.398 (2)
C8—C9	1.420 (3)	C21—C22	1.389 (3)
C8—H8	0.9500	C21—H21	0.9500
C9—C10	1.431 (3)	C22—C23	1.382 (3)
C10—C11	1.528 (2)	C22—H22	0.9500
C11—C12	1.507 (3)	C23—C24	1.378 (3)
C11—C20	1.537 (2)	C24—C25	1.396 (3)
C11—H11	1.0000	C24—H24	0.9500
C12—C17	1.347 (3)	C25—H25	0.9500
C12—C13	1.477 (3)		
			
C17—O1—C1	117.88 (15)	C15—C14—H14B	108.9
C10—C1—O1	123.37 (16)	H14A—C14—H14B	107.7
C10—C1—C2	122.89 (18)	C18—C15—C14	110.08 (17)
O1—C1—C2	113.74 (17)	C18—C15—C19	109.41 (17)
C3—C2—C1	119.07 (18)	C14—C15—C19	109.66 (16)
C3—C2—H2	120.5	C18—C15—C16	109.79 (17)
C1—C2—H2	120.5	C14—C15—C16	107.30 (16)
C2—C3—C4	121.38 (17)	C19—C15—C16	110.58 (17)
C2—C3—H3	119.3	C17—C16—C15	113.05 (16)
C4—C3—H3	119.3	C17—C16—H16A	109.0
C5—C4—C3	121.86 (17)	C15—C16—H16A	109.0
C5—C4—C9	119.55 (19)	C17—C16—H16B	109.0
C3—C4—C9	118.58 (18)	C15—C16—H16B	109.0
C6—C5—C4	120.98 (18)	H16A—C16—H16B	107.8
C6—C5—H5	119.5	C12—C17—O1	122.17 (17)
C4—C5—H5	119.5	C12—C17—C16	126.17 (17)
C5—C6—C7	120.03 (19)	O1—C17—C16	111.65 (16)
C5—C6—H6	120.0	C15—C18—H18A	109.5
C7—C6—H6	120.0	C15—C18—H18B	109.5
C8—C7—C6	120.4 (2)	H18A—C18—H18B	109.5
C8—C7—H7	119.8	C15—C18—H18C	109.5
C6—C7—H7	119.8	H18A—C18—H18C	109.5
C7—C8—C9	121.35 (18)	H18B—C18—H18C	109.5
C7—C8—H8	119.3	C15—C19—H19A	109.5
C9—C8—H8	119.3	C15—C19—H19B	109.5
C8—C9—C10	122.59 (16)	H19A—C19—H19B	109.5
C8—C9—C4	117.64 (17)	C15—C19—H19C	109.5
C10—C9—C4	119.76 (18)	H19A—C19—H19C	109.5
C1—C10—C9	118.22 (16)	H19B—C19—H19C	109.5
C1—C10—C11	120.41 (16)	C21—C20—C25	118.86 (17)
C9—C10—C11	121.34 (16)	C21—C20—C11	119.82 (16)
C12—C11—C10	109.01 (15)	C25—C20—C11	121.32 (17)
C12—C11—C20	111.06 (14)	C20—C21—C22	121.22 (17)
C10—C11—C20	110.16 (15)	C20—C21—H21	119.4
C12—C11—H11	108.9	C22—C21—H21	119.4
C10—C11—H11	108.9	C23—C22—C21	118.53 (18)
C20—C11—H11	108.9	C23—C22—H22	120.7
C17—C12—C13	118.09 (17)	C21—C22—H22	120.7
C17—C12—C11	123.37 (17)	C24—C23—C22	122.26 (18)
C13—C12—C11	118.54 (16)	C24—C23—Cl1	119.47 (14)
O2—C13—C12	120.53 (18)	C22—C23—Cl1	118.26 (16)
O2—C13—C14	121.94 (17)	C23—C24—C25	118.30 (17)
C12—C13—C14	117.47 (16)	C23—C24—H24	120.8
C13—C14—C15	113.33 (16)	C25—C24—H24	120.8
C13—C14—H14A	108.9	C24—C25—C20	120.83 (19)
C15—C14—H14A	108.9	C24—C25—H25	119.6
C13—C14—H14B	108.9	C20—C25—H25	119.6
			
C17—O1—C1—C10	−13.7 (3)	C17—C12—C13—O2	−177.76 (17)
C17—O1—C1—C2	166.34 (15)	C11—C12—C13—O2	1.7 (2)
C10—C1—C2—C3	−2.7 (3)	C17—C12—C13—C14	5.0 (2)
O1—C1—C2—C3	177.34 (17)	C11—C12—C13—C14	−175.55 (15)
C1—C2—C3—C4	1.7 (3)	O2—C13—C14—C15	146.05 (17)
C2—C3—C4—C5	179.68 (18)	C12—C13—C14—C15	−36.7 (2)
C2—C3—C4—C9	1.0 (3)	C13—C14—C15—C18	175.54 (16)
C3—C4—C5—C6	−177.85 (18)	C13—C14—C15—C19	−64.0 (2)
C9—C4—C5—C6	0.8 (3)	C13—C14—C15—C16	56.1 (2)
C4—C5—C6—C7	0.7 (3)	C18—C15—C16—C17	−166.12 (17)
C5—C6—C7—C8	−1.7 (3)	C14—C15—C16—C17	−46.5 (2)
C6—C7—C8—C9	1.3 (3)	C19—C15—C16—C17	73.1 (2)
C7—C8—C9—C10	−179.36 (18)	C13—C12—C17—O1	−174.47 (15)
C7—C8—C9—C4	0.1 (3)	C11—C12—C17—O1	6.1 (3)
C5—C4—C9—C8	−1.2 (3)	C13—C12—C17—C16	4.3 (3)
C3—C4—C9—C8	177.50 (17)	C11—C12—C17—C16	−175.15 (16)
C5—C4—C9—C10	178.32 (17)	C1—O1—C17—C12	11.2 (3)
C3—C4—C9—C10	−3.0 (3)	C1—O1—C17—C16	−167.72 (15)
O1—C1—C10—C9	−179.32 (16)	C15—C16—C17—C12	18.3 (3)
C2—C1—C10—C9	0.7 (3)	C15—C16—C17—O1	−162.83 (15)
O1—C1—C10—C11	−1.1 (3)	C12—C11—C20—C21	−66.9 (2)
C2—C1—C10—C11	178.86 (17)	C10—C11—C20—C21	54.0 (2)
C8—C9—C10—C1	−178.37 (17)	C12—C11—C20—C25	113.16 (19)
C4—C9—C10—C1	2.1 (3)	C10—C11—C20—C25	−125.96 (18)
C8—C9—C10—C11	3.5 (3)	C25—C20—C21—C22	0.5 (3)
C4—C9—C10—C11	−176.02 (16)	C11—C20—C21—C22	−179.52 (16)
C1—C10—C11—C12	16.0 (2)	C20—C21—C22—C23	−0.9 (3)
C9—C10—C11—C12	−165.92 (16)	C21—C22—C23—C24	0.6 (3)
C1—C10—C11—C20	−106.13 (19)	C21—C22—C23—Cl1	−178.22 (14)
C9—C10—C11—C20	72.0 (2)	C22—C23—C24—C25	0.2 (3)
C10—C11—C12—C17	−18.8 (2)	Cl1—C23—C24—C25	178.95 (15)
C20—C11—C12—C17	102.80 (19)	C23—C24—C25—C20	−0.6 (3)
C10—C11—C12—C13	161.81 (15)	C21—C20—C25—C24	0.3 (3)
C20—C11—C12—C13	−76.6 (2)	C11—C20—C25—C24	−179.72 (17)

### Hydrogen-bond geometry (Å, °)

**Table:**

*D*—H···*A*	*D*—H	H···*A*	*D*···*A*	*D*—H···*A*
C24—H24···O2^i^	0.95	2.56	3.376 (2)	144

Symmetry codes: (i) −*x*+1, −*y*+1, −*z*+2.
